# Optogenetic Interrogation of Circuits Following Neurotrauma

**DOI:** 10.3389/fnmol.2021.803856

**Published:** 2021-12-16

**Authors:** Steven Ceto, Grégoire Courtine

**Affiliations:** ^1^Center for Neuroprosthetics and Brain Mind Institute, School of Life Sciences, Swiss Federal Institute of Technology (EPFL), Lausanne, Switzerland; ^2^Department of Clinical Neuroscience, Lausanne University Hospital (CHUV) and University of Lausanne (UNIL), Lausanne, Switzerland; ^3^Defitech Center for Interventional Neurotherapies (.NeuroRestore), EPFL/CHUV/UNIL, Lausanne, Switzerland; ^4^Department of Neurosurgery, Lausanne University Hospital (CHUV), Lausanne, Switzerland

**Keywords:** optogenetics, calcium imaging, cell type specificity, all-optical, circuit dissection, stroke, spinal cord injury, graft

## Abstract

Biological and engineering strategies for neural repair and recovery from neurotrauma continue to emerge at a rapid pace. Until recently, studies of the impact of neurotrauma and repair strategies on the reorganization of the central nervous system have focused on broadly defined circuits and pathways. Optogenetic modulation and recording methods now enable the interrogation of precisely defined neuronal populations in the brain and spinal cord, allowing unprecedented precision in electrophysiological and behavioral experiments. This mini-review summarizes the spectrum of light-based tools that are currently available to probe the properties and functions of well-defined neuronal subpopulations in the context of neurotrauma. In particular, we highlight the challenges to implement these tools in damaged and reorganizing tissues, and we discuss best practices to overcome these obstacles.

## Introduction

Neurotrauma such as brain injuries, stroke, and spinal cord injury (SCI) scatter the finely organized network of circuits that produce behaviors, causing devastating cognitive and/or sensorimotor impairments. Many biological and engineering strategies seek to repair these circuits to enhance functional recovery from neurotrauma. Understanding the consequences of neurotrauma and the mechanisms underlying repair strategies requires tools to visualize the neuroanatomical and functional properties of circuits in the brain and spinal cord. Optogenetics has triggered a paradigm shift in the resolution of these evaluations. Traditionally, electrical stimulation and recording methods have been the main tools for studying broadly defined circuits and pathways.

However, the results of these experiments are difficult to interpret, since these methods cannot distinguish between the various subpopulations of neurons that constitute the brain and spinal cord. Optogenetics leverages the expression of genetically engineered proteins to target specific neuronal subpopulations. These methods enable activation, silencing, and recording of neural activity with light in precisely-defined neurons, which is not possible with electricity-based methods (Fenno et al., 2011; Grienberger and Konnerth, 2012; Emiliani et al., 2015; Kim et al., 2017). Optogenetic tools are genetically encoded molecules, such as those derived from the opsin family of light-sensitive proteins, that drive or silence the activity of genetically targeted neurons by light with millisecond-timescale (Fenno et al., 2011). However, this terminology does not have to be restricted to the modulation of neuronal activity, since optical imaging of genetically targeted neurons has become a prominent methodology to record the neural activity from well-defined neuronal subpopulations. For example, calcium imaging uses calcium ion dynamics to visualize neural activity. Genetically encoded calcium indicators (GECIs) are composed of a fluorescent protein linked to a calcium binding domain (Grienberger and Konnerth, 2012). These molecules increase in fluorescence upon binding to calcium ions, which are released into the cytoplasm in large waves upon action potential firing. Calcium imaging can therefore be used to visualize neural activity. Voltage indicators are membrane-bound molecules that change in fluorescence with membrane voltage at much faster speeds than calcium indicators (Inagaki and Nagai, 2016). They allow detection of both sub- and suprathreshold neuronal activity at near physiological timescales, but the fluorescence changes are much smaller in amplitude, necessitating highly sensitive and fast optical recording equipment. Genetically encoded and synthetic dye versions of both calcium and voltage indicators are available. Here, we consider optogenetics as multifaceted methods to activate, silence, and record genetically targeted neurons.

The use of optogenetics to assess the properties and function of neurons located in the brain and spinal cord tissues that have undergone trauma brings unique challenges. In this mini-review, we summarize a selection of studies that have addressed these challenges in the context of neurotrauma, both untreated and following molecular intervention. Additionally, we highlight the use of optogenetics to interrogate the functional integration of cell grafts with endogenous circuitry. Finally, we provide a guide to apply optical methodologies for the understanding of damaged, reorganizing, and repaired circuits following neurotrauma.

## Optogenetic Interrogation of Neurons in The Brain After Neurotrauma

**Figure 1:**
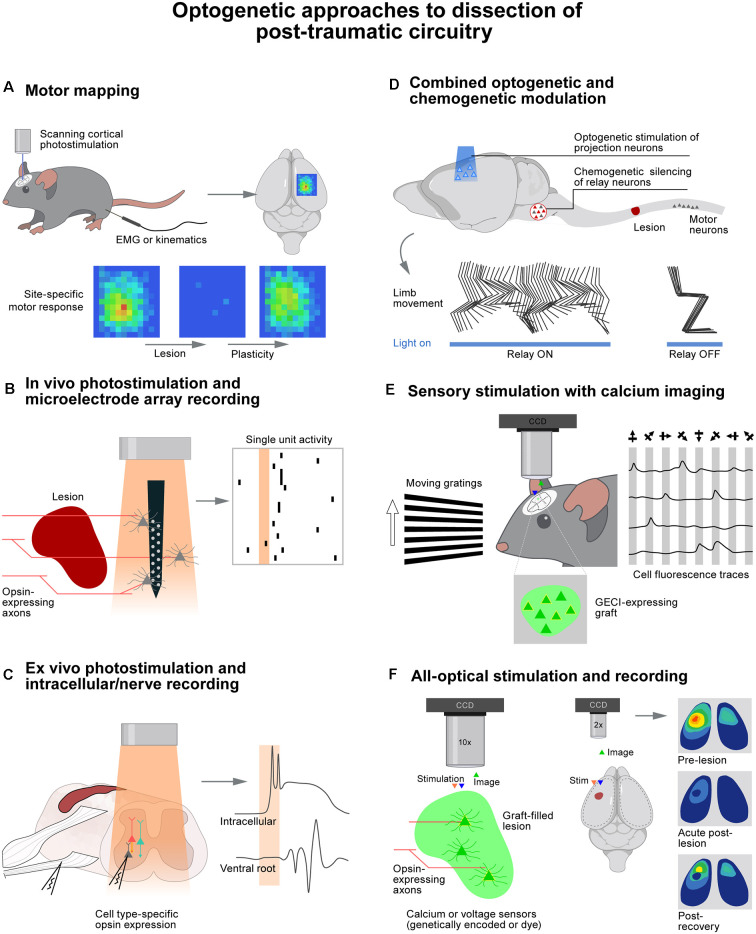
Optogenetic approaches to dissection of post-traumatic circuitry. **(A)** Photostimulation of the cortex over a grid of sites while recording muscle responses or limb movements reveals motor plasticity following trauma to the brain or spinal cord. **(B)** Microelectrode arrays do minimal damage to perilesional tissue, allowing recording of responses to noninvasive photostimulation of sprouted or regenerated axons. **(C)** *Ex vivo* preparations enable precise control of drug and ion concentrations with clear access to obtain intracellular recordings. **(D)** Temporally precise optogenetic stimulation combined with noninvasive chemogenetic silencing of neuronal populations is a powerful approach to dissecting circuits critical for functional recovery after injury. **(E)** The activity of genetically defined neuronal populations such as cell grafts can be monitored during sensory stimulation (or motor behavior) to assess functional circuit integration. **(F)** All optical approaches allow simultaneous genetically defined stimulation and recording of large numbers of cells. Wavelengths of light used for stimulation and recording must be well separated.

The ability of the central nervous system to reorganize following damage provides hope for functional recovery, but, at the same time, introduces specific challenges for assessing the reorganizing or newly formed circuits. Optogenetic approaches have proven to be well suited to overcome these challenges (Table 1), especially in the context of highly damaged tissues, since photostimulation and imaging methodologies allow interrogation of the circuits embedded in these tissues with minimal additional insults. For example, optogenetics has been used to study cortical neurons in the hyperacute phase after stroke. The expression of channelrhodopsin-2 (ChR2) in cortical projection neurons enabled the assessment of neuronal excitability during and immediately after the infarct (Chen et al., 2012). In this study, they measured electroencephalogram and local field potential activity that occurred spontaneously, in response to sensory stimulation, and following direct optogenetic activation of cortical neurons. They could demonstrate that neurons located in the region of infarct remain excitable during ischemia and reperfusion, but that synaptic inputs to these neurons are largely silent. Furthermore, output from cortical projection neurons was found to be less affected by ischemia compared to sensory processing (Xie et al., 2013). To study the changes over the entire cortex during recovery following ischemia, the same group implemented an all-optical approach combining ChR2 photostimulation and voltage-sensitive dye imaging (Figure 1F; Lim et al., 2014). They revealed an asymmetry in the recovery of neuronal activity in the peri-infarct regions. Specifically, the regions exhibiting less connectivity with the infarct core before stroke recovered better than the areas with denser connectivity to the core. In a model of small vessel disease using microspheres to generate microinfarcts, the mesoscopic activity of excitatory neurons was monitored over the entire cortex using transcranial calcium imaging (Balbi et al., 2019). This study targeted excitatory neurons by expressing the calcium indicator GCaMP6 in glutamatergic neurons. They found a surprising lack of change in functional connectivity within the cortex despite deficits in motor function in microsphere-injected mice—perhaps exposing limitations of a low-resolution imaging approach to examine small-scale pathologies.

**Table 1:** List of studies utilizing optogenetic methods to interrogate reorganizing circuitry after acute trauma and intervention.

Optogenetic modality	Other modality	Lesion	Circuit	Optogenetic effectors	References
Actuation	EEG, MEA (optrode), IOS imaging	Cortical stroke	CTX → CTX	ChR2	Chen et al. (2012)
	EEG, EMG	Cortical stroke	CTX → CTX; CTX → forelimb motor neurons	ChR2	Xie et al. (2013)
	VSD imaging	Cortical stroke	CTX → CTX	ChR2	Lim et al. (2014)
	Single-electrode EP	TBI (CTX)	CTX → CTX	ChR2	Adams et al. (2018)
	EMG	TBI (CTX)	CTX → forelimb spinal MNs	ChR2	Nguyen et al. (2021)
	Forelimb movement	SCI	CTX → forelimb	ChR2	Hollis et al. (2016)
	Limb movement	SCI	CTX → forelimb, hindlimb	ChR2	Hilton et al. (2016)
	EMG, c-Fos	SCI	CTX → forelimb spinal MNs	ChR2	Qian et al. (2019)
	EMG	SCI	Spinal INs → diaphragmatic spinal MNs	ChR2	Alilain et al. (2008)
	Limb movement	Pyramidotomy	CTX → forelimb, hindlimb	ChR	Jin et al. (2015)
	EMG	SCI	Spinal axons → forelimb spinal MNs	ChR2	Chen J. Y. et al. (2021)
	MEA	Pyramidotomy	CTX → spinal neurons	ChR2	Jayaprakash et al. (2016)
	MEA	SCI	CTX → spinal neurons	ChR2	Sun et al. (2019)
	Limb movement, chemogenetic silencing	SCI	CTX →vGi → hindlimb MNs	ChR2	Asboth et al. (2018)
	ICR, VRR	SCI	V3 spinal INs → spinal MNs	ChR2, Arch3	Lin et al. (2019)
	EMG, VRR, DRS	SCI	DRG → spinal INs → spinal MNs	ChR2, eNpHR3	Bellardita et al. (2017)
	Hindlimb movement, EMG	SCI	DRG → spinal INs → spinal MNs	Jaws, ChrimsonR	Kathe et al. (2021)
	ICR, behavior	Chemical lesion (striatum)	Graft → striatum	eNpHR3	Steinbeck et al. (2015)
	ICR, drug application	Chemical lesion (substantia nigra)	Striatum → graft, graft → graft; graft → host	ChR2, NpHR	Tønnesen et al. (2011)
	Single-electrode EP, ICR	Cortical stroke	Thalamus → cortical graft	ChR2	Tornero et al. (2017)
	ICR	N/A	HPC → graft	ChR2	Avaliani et al. (2014)
	ICR	SCI	CTX → spinal cord graft	ChR2	Kadoya et al. (2016)
	MEA	SCI	CTX → spinal neurons; CTX → spinal cord graft(?)	ChR2	Jayaprakash et al. (2019)
	fMRI, MEA	N/A	Striatal graft → striatum, CTX, HPC, septal nuclei	ChR2	Byers et al. (2015)
	MEA	Aspirative lesion in CTX	Cortical organoid graft → CTX	ChR2	Mansour et al. (2018)
					
Recording	Whisker stimulation	Microinfarct	Whisker → CTX	GCaMP6s, GCaMP6f	Balbi et al. (2019)
	Drug application	TBI	CTX → CTX	GCaMP6f	Nguyen et al. (2021)
	Blast injury	TBI	HPC → HPC	GCaMP6f	Hansen et al. (2018)
	DRS, VRR	SCI	DR → Spinal INs → spinal MNs	GCaMP3	Bellardita et al. (2017) and Marcantoni et al. (2020)
	N/A	Aspirative lesion in CTX	Cortical organoid graft → graft	jRGECO1a	Mansour et al. (2018)
	Visual stimuli	N/A	CTX → cortical graft	GCaMP6s	Zheng et al. (2021)
	Visual stimuli	Photoactivation-induced apoptotic cell death (CTX)	CTX, Thalamus → cortical graft	GCaMP6 s, Twitch2B	Falkner et al. (2016)
Stimulation and recording (simultaneous)	Light touch, tail pinch	SCI	CTX → spinal cord graft; graft → graft; graft → spinal neurons; sensory neurons → graft	ChrimsonR, GCaMP6f	Ceto et al. (2020)

Optogenetic methods have also been used to study neuronal circuits following traumatic brain injury (TBI). Using photostimulation of cortical neurons located in the vicinity of regions receiving repeated TBIs, it was possible to confirm that functional deficits correlate with reduced cerebral blood flow and the presence of astrogliosis (Adams et al., 2018). Another study employed optogenetic motor mapping and calcium imaging to characterize the early impact of mild closed head TBI on the size of motor maps and neuronal population dynamics (Nguyen et al., 2021). They detected fewer and slower calcium transients post-TBI, which were associated with changes in glutamate receptor function. Comparable declines in fast calcium transients were observed with GCaMP6 imaging of hippocampal neurons following mild blast injury (Hansen et al., 2018).

Reorganization of cortical projection neurons after SCI has been elegantly examined using optogenetics. Using photostimulation of motor cortex projection neurons in Thy1-ChR2 mice (Figure 1A), it was possible to expose a massive remapping of cortical output following the interruption of the dorsal column lesion at the C5 level (Hollis et al., 2016). Over the course of recovery, forelimb-related motor maps progressively expanded into areas that were exclusively representing hindlimb movements before the injury. This reorganization of motor cortex output was exaggerated in mice with a conditional knockout of Ryk, a Wnt receptor protein known to be repulsive to axon growth. This enhanced reorganization was thus interpreted as a sign of increased neuronal reorganization after injury in the absence of this receptor. Thy1-ChR2 mice also enabled dissecting the role of specific components of the corticospinal tract. Following the interruption of the dorsal column, the majority of corticospinal tract fibers are interrupted. However, a subset of these fibers project in the dorsolateral column and are thus spared by the injury. Photostimulation of motor cortex neurons was therefore used to probe the role of these spared projections (Hilton et al., 2016). Optogenetics provides the opportunity to stimulate different cortical regions by laser scanning of the optic fiber tip above the cortex instead of repeatedly inserting and withdrawing a stimulating electrode. This methodology supported the generation of motor output maps within a few minutes in the hyperacute stage of injury. The same approach was used to ask whether the red nucleus is able to relay cortical commands to the spinal cord following a contusion SCI that largely spares rubrospinal projections. The combination of optically-enabled electrophysiology, anatomical connectivity, and activity-dependent markers provided evidence suggesting that rubrospinal projections contribute to the recovery of cortically-evoked hindlimb movements (Qian et al., 2019).

We employed a comparable strategy to study the mechanisms enabling the motor cortex to regain control of walking when applying electrochemical neuromodulation to the lumbar spinal cord after a severe contusion SCI (Asboth et al., 2018). Using photostimulation of motor cortex projection neurons with graded intensities, we were able to demonstrate that electrochemical neuromodulation instantly enables the motor cortex to regain adaptive control over the activity of otherwise paralyzed muscles. Whole-brain and spinal cord anatomical quantifications revealed that the SCI interrupted all the corticospinal projections below the injury, but systematically spared a subset of reticulospinal projections. Chemogenetic silencing of glutamatergic neurons located in the ventral gigantocellular reticular nuclei instantly suppressed the cortical control of walking (Figure 1D). These experiments revealed that cortico–reticulo–spinal circuit reorganization enables functional recovery of walking after severe contusion SCI.

This ensemble of experiments illustrates the precision enabled by optogenetics to uncover the contribution of specific cortical neurons and descending pathways in mediating complex behaviors and how their reorganization can contribute to recovery after neurotrauma.

## Optogenetic Interrogation of The Spinal Cord After Neurotrauma

Optogenetics has also been deployed to interrogate neurons and pathways in the spinal cord. For example, early experiments leveraged ChR2 to demonstrate that photostimulation of the phrenic motor nucleus below a cervical SCI could restore respiratory diaphragmatic motor activity (Alilain et al., 2008). This rhythmic activity continued at a lower amplitude up to 24 h after long periods of photostimulation, suggesting that the stimulation had induced activity-dependent reorganization of the stimulated neurons.

Light-sensitive channels can be expressed along the length of axons and within pre-synaptic terminals. Therefore, applying photostimulation over the spinal cord allows the interrogation of residual projections after SCI. This strategy has been used to activate axons from spared corticospinal tract projections *via* a fiber positioned above the surface of the spinal cord. As such, several groups were able to investigate whether therapeutic interventions targeting the corticospinal tract promoted reorganization or regeneration of this tract. For instance, co-deletion of the PTEN and SOCS3 genes was shown to promote the growth of new corticospinal projections to the contralateral hemicord following unilateral pyramidotomy (Jin et al., 2015). Accordingly, photostimulation of corticospinal tract fibers within the cervical spinal cord elicited bilateral forelimb movements, whereas the same stimulation only triggered ipsilateral forelimb movements in wild-type mice. Another example comes from a therapy based on the delivery of anti-inflammatory interleukin-10 following a lateral hemisection of the cervical spinal cord. It was found that photostimulation of projections below the injury-induced robust responses in forelimb muscles (Chen J. Y. et al., 2021).

The impact of neuroregenerative treatments on the reorganization of supraspinal projections in the spinal cord has also been examined using silicon multielectrode recording arrays combined with photostimulation (Figure 1B; Jayaprakash et al., 2016; Sun et al., 2019). One study examined the role of corticospinal tract projections after a pyramidotomy and over-expression of the transcription factor Sox11 along with ChR2 in motor cortex projection neurons. They found that photostimulation of residual and reorganizing corticospinal projections induced postsynaptic responses in neurons located in the ventral spinal cord (Jayaprakash et al., 2016).

Spinal circuits involved in detrimental outcomes after neurotrauma such as spasticity have also been dissected with optogenetics. Genetically-defined subpopulations of spinal neurons can be readily interrogated using photostimulation (Figure 1C). For example, V3 neurons were targeted using Cre-dependent expression of ChR2 or Arch3 in Sim1^ON^ cells. This excitatory population of commissurally projecting neurons could thus be stimulated or inhibited in *ex vivo* spinal cord preparations from mice with chronic SCI that had developed spasticity (Lin et al., 2019). Photostimulation of V3 neurons alone triggered spasm-like activity similar to that evoked by dorsal root stimulation. Likewise, photoinhibition of V3 neurons reduced the amplitude of spasms. The same approach was implemented *in vivo*. Suprathreshold photostimulation of V3 neurons induced tail spasms, whereas subthreshold light trains overwhelmed V3 neurons and suppressed ongoing spasms. The distinct roles of excitatory and inhibitory spinal interneuron populations in spasm generation and persistence were also dissected using a combination of optogenetic modulation and additional calcium imaging. These experiments documented the spread of activity through various subpopulations of neurons in response to dorsal root stimulation (Bellardita et al., 2017; Marcantoni et al., 2020).

Optogenetic modulation of spinal circuits in behaving animal models requires specialized optoelectronic devices. Indeed, the extensive displacements of spinal tissues relative to the vertebral column during unconstrained behaviors prevent the use of penetrating optical fibers or other classic approaches to deliver light throughout the spinal cord (Montgomery et al., 2015; Park et al., 2015; Lu et al., 2017; Kathe et al., 2021). Various devices have overcome the challenges of spinal cord optogenetics. For example, we recently engineered a wireless optoelectronic implant that enables long-term photostimulation of any neurons and pathways embedded in the spinal cord while untethered mice can behave freely without any constraint. This deeply-penetrating photostimulation is achieved using the activation of red-shifted opsins since this wavelength of light is less attenuated by spinal tissue. Photostimulation is delivered through micro-LEDs that are integrated within a soft, stretchable carrier implanted over the dura mater (Kathe et al., 2021). This implant enabled us to study the role of proprioceptive feedback circuits in the production of walking enabled by serotonergic pharmacotherapy after complete SCI. Unexpectedly, sudden optogenetic silencing of proprioceptive neurons led to an increase in step height over a succession of steps, before causing the mice to collapse. This observation highlights the complex dynamics of spinal circuits and the importance of targeted and precisely timed neuromodulation strategies to evaluate the contribution of neurons and circuits to the production of complex behavior.

## Optical Interrogation of Grafted Neurons and Connectivity with Host Neurons

Cell transplantation therapies aim to establish an intimate integration of grafted cells within the host environment. Consequently, interrogation of the synaptic connectivity between grafted and host neurons benefits greatly from the ability to target genetically defined neuronal populations to achieve optical recording and stimulation. Despite a long history of dopaminergic cell therapies showing improved behavior in preclinical models of Parkinson’s disease, it was not until it became possible to silence grafted neurons with halorhodopsin that the beneficial role of these grafts could be established unequivocally (Steinbeck et al., 2015). Indeed, photoinhibition of human embryonic stem cells grafted into striatal lesion sites immediately and reversibly reinstated motor deficits alleviated by this graft, thereby confirming the beneficial functional integration of grafts with host motor circuits. These results were supported by prior studies that had dissected the connectivity involved in host-graft communication using acute slice preparations combined with optogenetic activation and silencing of host and graft neurons (Tønnesen et al., 2011). Optogenetic stimulation of specific ensembles of host afferents based on their typology, origin, or ending is a useful strategy to uncover functional inputs to graft neurons. In a model of cortical stroke, photostimulation of ChR2-expressing thalamic axons at the level of the cerebral cortex elicited excitatory postsynaptic responses in human induced pluripotent stem cells (hiPSCs) transplanted within and in the periphery of the injured region (Tornero et al., 2017). Likewise, photostimulation of host hippocampal neurons was able to drive postsynaptic action potentials in inhibitory hiPSCs that were aimed at controlling seizure activity (Avaliani et al., 2014). In the spinal cord, ChR2-expressing corticospinal axons have been shown to drive responses in neural progenitor cells (NPC) grafted in sites of SCI using acute slice preparations (Kadoya et al., 2016; Ceto et al., 2020) and possibly *in vivo* (Jayaprakash et al., 2019).

The low number of cells that can be recorded with classical whole-cell patch techniques has compelled investigators to implement functional imaging techniques, sometimes in combination with optogenetics, to probe the function of grafts. For instance, functional magnetic resonance imaging was used to monitor responses across the hippocampus and remote cortical areas when photostimulating ChR2-expressing hiPSCs transplanted in the dorsal striatum of rats (Byers et al., 2015). Calcium imaging was used to image both graft and host activity at single cell resolution in a mouse model of SCI (Ceto et al., 2020). This all-optical approach utilized the red-shifted channelrhodopsin, named ChrimsonR, for stimulation of host corticospinal or graft axons while imaging neuronal activity *via* GCaMP6 expression in graft or host neurons (Figure 1F). It was found that graft neurons were activated in distinct assemblies with highly correlated spontaneous activity, demonstrating the formation of complex webs of interconnected cells. These networks appeared to integrate with host systems since photostimulation of corticospinal tract axons activated distinct assemblies of graft neurons. Sensory stimuli also elicited calcium responses in grafts *in vivo*. Moreover, photostimulation of graft axons evoked neuronal responses in host circuits below the injury, indicating the likely formation of graft-mediated neuronal relays across the site of SCI. Finally, the neocortex has proven to be a particularly amenable site for calcium imaging of graft activity due to its ease of optical access (Figure 1E). Accordingly, it was possible to show that transplanted brain organoids displayed spontaneous calcium transients in the retrosplenial cortex (Mansour et al., 2018), and visual stimuli evoked calcium responses in inhibitory interneuron (Zheng et al., 2021) and embryonic NPC grafts (Falkner et al., 2016) in the visual cortex.

This series of studies illustrated the unique advantages of optical recording and stimulation methodologies to study the integration and role of graft cells in the nervous system.

## Limitations of Optical Approaches

Before designing any optogenetics-based experiment, it is important to understand the limitations and potential caveats associated with these techniques. Although it is useful to be able to modulate genetically defined neuronal populations, it is by no means naturalistic. Simultaneous activation or suppression of opsin-expressing neurons, regardless of the relative proportion of targeted cells, would never occur during the natural operations of the nervous system. Consequently, the responses evoked by the synchronous photostimulation of these neurons may lead to a much different type and level of output than would normally occur. This is especially true when axon terminals are stimulated rather than cell bodies since many neural populations intercalated between the cell body and stimulated regions are then left out of the picture. This may or may not be desirable, depending on the experimental question. Alteration of neuronal properties is another concern for long-term experiments. Indeed, long-term expression of membrane-bound opsins such as ChR2 should be avoided if possible, as a high abundance of these molecules in axons can change their morphology and potentially their synaptic function, particularly if expression begins during development or in graft cell culture (Miyashita et al., 2013).

Optogenetics effectively modulates the activity of neurons over a broad range of opsin expression levels. In contrast, optical recordings require an optimal titration of indicators to produce a detectable yet unsaturated signal. Compounding this challenge is the requirement for clear optical access to the targeted regions. Yet, many regions of the central nervous system are prone to hemorrhage and immune cell invasion, especially during the days and weeks that follow a neurotrauma. In the case of calcium imaging, the targeted cells may display abnormal calcium dynamics if directly involved in the studied neuropathology, and calcium indicators themselves may alter these dynamics due to their calcium buffering properties (Steinmetz et al., 2017; Singh et al., 2018). Finally, the temporal resolution of calcium imaging is limited to 10 s of ms, whereas the underlying neuronal activity occurs at sub-millisecond timescales. Although efforts have been made to infer the composition of spike trains responsible for the generated calcium activity in highly reproducible settings (Carrillo-Reid et al., 2008; Giovannucci et al., 2019), this type of approach is not feasible in the variable and dynamic context of neurotrauma.

## Selecting The Optimal Optical Strategy

The significant time and resources necessary to establish a new optogenetic experiment dictates careful consideration of the biological question at hand. Is a simple binary result sufficient? Or is a detailed analysis of circuit dynamics required? The temporal resolution necessary to ask these questions will determine the type of modulation and recording modalities that are appropriate. If precise timing of modulation is critical, traditional channel-based opsins are ideal. Recent channelrhodopsin variants can drive firing rates up to several hundred hertz (Mager et al., 2018), or be used noninvasively for deep (up to 7 mm) targets (Chen R. et al., 2021). If long-term (minutes to hours) modulation is desired, G-protein-coupled receptor-based opsins (opto-XRs), step function opsins, or chemogenetic actuators are more appropriate (Eickelbeck et al., 2019; Keifer et al., 2020). Conveniently, many opsin constructs are packaged with linked fluorescent proteins or tags that, when bound to the plasma membrane along with the opsin, enable robust anatomical tracing even of narrow, regenerating axons.

When monitoring neural activity, it is essential to define the necessary temporal resolution. For example, if experiments require distinguishing between mono- and polysynaptic responses, optogenetically enabled cell-specific electrical recordings must be preferred since they provide accurate response latencies when calcium transients are delayed by tens to hundreds of milliseconds. This methodology is termed optogenetic tagging (Lima et al., 2009; Caggiano et al., 2018; Kremer et al., 2020). It consists of recording electrical activity from cells that express excitatory opsins, which are thus identified based on their consistent low-latency and low-jitter responses to photostimulation. Minimally invasive slim microelectrode arrays provide the advantage of high temporal precision while enabling recordings from genetically defined neuronal populations. Depending on the location of the relevant neurons, such arrays may be the only viable solution for *in vivo* recordings. GRIN lenses, microprisms, and fiber photometry have been used to access deep regions of the intact brain that cannot be imaged through superficial window preparations (Ghosh et al., 2011; Andermann et al., 2013; Cai et al., 2016; Pisano et al., 2019; Wang et al., 2021). However, the large size of these optical probes may prevent smooth insertion into post-traumatic tissue toughened by scarring and prone to bleeding. Furthermore, dynamic calcium signals are often not observed until weeks after implantation, suggesting that the insertion of optical probes damages the surrounding circuits. This limitation not only prevents acute recording entirely but may also confound results of experiments examining a pre-existing pathology. Static signals are not as common in healthy slice preparations, indicating a role of the immune system in saturating calcium signals acutely *in vivo*.

While many of the studies reviewed here utilized optogenetic tools expressed in broad or poorly defined neuronal populations, such as in Thy1-ChR2 mice, future experiments should take advantage of intersectional approaches to target precise subpopulations with unique molecular signatures and/or projection patterns. This can be facilitated by the use of reporter alleles under the control of multiple recombinases (e.g., Cre and Flp) whose expression can be driven by distinct promoters (Madisen et al., 2015). Transgenic and viral approaches can be used separately or in combination depending on the anatomical specificity required. Identifying the neuronal subpopulations mediating functional changes following neurotrauma will enable the development of precision therapies that act on the most relevant circuit components while minimizing off-target effects.

## Conclusions

Optogenetics has transformed the field of neuroscience. These methodologies have enabled unprecedented precision to interrogate the role and function of neurons within the realm of neurotrauma, albeit the implementation of these tools comes with a unique set of challenges. Harnessing the full power of these methods requires a careful design of experiments in a way that takes advantage of the specificity of the employed tools while controlling for potential side effects of both their expression and activation in the presence of existing tissue damage. While the current toolbox for optogenetic modulation and recording has expanded widely over the past decade, it continues to undergo rapid developments that promise the availability of more precise and less invasive methods for optical circuit interrogation.

## Author Contributions

SC conceptualized and wrote the original draft and prepared the figure and table. SC and GC edited and revised the manuscript. GC supervised the work. All authors contributed to the article and approved the submitted version.

## Conflict of Interest

The authors declare that the research was conducted in the absence of any commercial or financial relationships that could be construed as a potential conflict of interest. The handling editor declared a past co-authorship with one of the authors GC.

## Publisher’s Note

All claims expressed in this article are solely those of the authors and do not necessarily represent those of their affiliated organizations, or those of the publisher, the editors and the reviewers. Any product that may be evaluated in this article, or claim that may be made by its manufacturer, is not guaranteed or endorsed by the publisher.

## Abbreviations

EEG: electroencephalogram
MEA: multi-electrode array
IOS: intrinsic optical signal
EMG: electromyogram
VSD: voltage-sensitive dye
EP: extracellular potential
ICR: intracellular recording
VRR: ventral root recording
DRS: dorsal roots stimulation
fMRI: functional magnetic resonance imaging
TBI: traumatic brain injury
SCI: spinal cord injury
CTX: cortex
MN: motor neuron
vGi: ventral gigantocellular reticular nucleus
DRG: dorsal root ganglia
IN: interneuron
HPC: hippocampus.
